# Experimental and modeling analyses of COD removal from industrial wastewater using the TiO_2_–chitosan nanocomposites

**DOI:** 10.1038/s41598-022-15387-0

**Published:** 2022-06-30

**Authors:** Shahin Heydari Orojlou, Saadat Rastegarzadeh, Behrooz Zargar

**Affiliations:** Department of Chemistry, Faculty of Sciences, Shahid Chamran University of Ahvaz, Ahvaz, Iran

**Keywords:** Chemical engineering, Nanoscale materials

## Abstract

In the present study, titanium oxide (TiO_2_) nanoparticles, chitosan, and several nanocomposites containing different mass dosages of TiO_2_ and chitosan have been applied as the adsorbent for COD removal from the industrial wastewater (Bouali Sina Petrochemical Company, Iran). The FESEM, XRD, and FTIR tests have been employed to characterize TiO_2_ nanoparticles, chitosan, and fabricated nanocomposites. Then, the effect of adsorption parameters, including TiO_2_–chitosan mass ratio (1:1, 1:2, and 2:1), adsorbent content (0.25–2.5 g), temperature (20–50 °C), pH (3–11), solution volume (100–500 mL), and contact time (30–180 min) on the COD reduction has also been monitored both experimentally and numerically. The Box–Behnken design of the experiment approves that TiO_2_–chitosan (1:1), adsorbent content of 2.5 g, temperature = 20 °C, pH 7.4, solution volume of 100 mL, and contact time = 180 min are the condition that maximizes the COD removal (i.e., 94.5%). Moreover, the Redlich–Peterson and Pseudo-second order models are the best isotherm and kinetic scenarios to describe COD removal’s transient and equilibrium behaviors. The maximum monolayer COD adsorption capacity of the TiO_2_–chitosan nanocomposite is 89.5 mg g^−1^. The results revealed that the industrial wastewater COD is better to remove using the TiO_2_–chitosan (1:1) at temperature = 20 °C.

## Introduction

The amount of oxygen required to oxidize organic pollutants in wastewater is defined as either COD (chemical oxygen demand) or BOD (biological oxygen demand)^1^. It is possible to employ chemical^2^, physical^2^, and biological^3^ scenarios, such as adsorption^4,5^, nano-adsorption^6^, membrane^7^, ion exchange, electrocoagulation^8^, bio-flocculation^9^, sewage sludge^10,11^, and filtration^12,13^ for the treatment of waste streams. Indeed, those separation processes that utilize the solid porous materials (i.e., adsorption) are among the most popular techniques due to their economic/operational features and a high achievable removal efficiency^14–16^. Generally, the advantages of adsorption process relative to other methods are: high performance, low cost, wide pH ranges, and easy operation. In the other hand, the waste product and low selectivity are some of the main disadvantages of adsorption process^17^.

Now a day, nano-scale solid materials have successfully improved properties of working fluids^18,19^, alloys^20,21^, and polymers^22^, efficiency of solar collectors^23^, and performance of wastewater treatment processes^24^. Keshtkar et al. utilized the synthesized alumina nanoparticles with different specific surface areas to adsorb nickel ions from synthetic wastewater^24^. Esmaeili-Faraj et al. studied the desulfurization of an actual diesel fuel sample by applying the alumina/polymer nanocomposite from numerical and experimental perspectives^25^.

The chitosan-based nanocomposites have been widely used for water/wastewater treatment^26,27^. This popularity is associated with the chitosan’s low cost and its amino or hydroxyl functional groups. Chung examined the applicability of chitosan with various deacetylation degrees for treating aquaculture wastewater^28^. The optimum COD removal of 69.7% has been reported for the chitosan with a 98% deacetylation degree. Dionisi et al. inspected the impact of chitosan adsorbent and pH on the pollutants’ elimination from the pot ale wastewater^29^. Thirugnanasambandham and Sivakumar focused on the zinc oxide-chitosan nanocomposite to efficiently treat the milk processing industry wastewater^30^. It has been reported that COD and turbidity can be reduced by applying the zinc oxide-chitosan nanocomposite. The adsorption efficiency of Chitosan-Citral Schiff for the treatment of a dairy industry’s wastewater was studied by Tsaneva et al.^31^. The maximum COD removal efficiency was approximately 35.3% under the optimum condition. Ligaray et al. studied the applicability of the bentonite-chitosan composite for COD removal from an industrial wastewater stream containing an initial COD concentration of 1348 ppm^32^. The maximum COD removal of 73.34% has been achieved at the optimum condition. The kinetic of removal of heavy metals (Copper, cadmium and chromium) from wastewater using chitosan based adsorbents have been studied by Prakash et al.^33–35^.The results show that that pseudo second order kinetic model correlates better with the experimental data^33–35^.

Titanium dioxide (TiO_2_) nanoparticles are non-toxic, photochemically stable, and possess a strong oxidation ability^36^. The TiO_2_ nanoparticles have been extensively utilized as either photocatalyst or adsorbent for the COD removal from wastewaters^37,38^. Belessi et al. examined the simultaneous removal/adsorption of COD and reactive red 195 from aqueous solutions employing the TiO_2_ nanoparticle^39^. The photocatalytic removal efficiency of COD from sewage using the TiO_2_ catalyst has been studied by Toke and Ingale^40^. Goutam et al. synthesized the green TiO_2_ nanoparticles and investigated their performance for tannery wastewater treatment^41^. The results state that the fabricated green TiO_2_ nanoparticles remove 82.26% and 76.48% of the COD and Cr (VI) ion, respectively. Utilizing pure TiO_2_ nanoparticles as a photocatalyst for COD removal has several limitations, including inadequate UV (ultraviolet) irradiation, small oxidative performance, and high cost^42^. Improving the surface properties of nanoparticles by the co-adsorbent is a technique suggested to overcome the TiO_2_ limitations and increase its COD removal efficiency from wastewater. Rojviroon et al. applied the synthesized TiO_2_-activated carbon by the sol–gel method to remove the COD and dye from the landfill leachate^43^. Maleki et al. concentrated on the ethylene dichloride removal from wastewater using the TiO_2_–graphene catalyst^44^. Li et al. investigated the electrocatalytic characteristics of the fabricated TiO_2_–SiO_2_/GAC particle for COD removal^45^. Recently, the TiO_2_–chitosan nanocomposite has been employed to remove organic acids, heavy metals, and dyes. The degradation rate of Rhodamine B using the TiO_2_–chitosan nanocomposite was reported by Zhang et al.^46^. Chen et al. used the thiourea-modified chitosan–TiO_2_ nanocomposite for removing 2,4-dichlorophenol and Cd(II) ions from an aqueous solution^47^. Farzana and Meenakshi investigated the degradation of methylene blue, reactive red2, and Rhodamine B, by the TiO_2_–chitosan composite by measuring the solution COD^48^. Wibowo et al. compared the BOD and COD reduction ability of zeolite, TiO_2_–chitosan, and TiO_2_-bentonite composite^49^. Ali et al. used the TiO_2_–chitosan fibers supported zero-valent nanoparticles for the organic compound removal^50^. The ability of the ion-imprinted TiO_2_–chitosan adsorbent was also studied for nickel removal from aqueous solutions^51^. Tao et al. used the TiO_2_–chitosan hybrid film to absorb lead from aqueous solutions^52^. Nawi et al. examined the impact of operating parameters on the anionic dye (reactive red 4) removal capacity of the TiO_2_–chitosan nanocomposite^53^. The efficiency of TiO_2_–chitosan nanofibers for metal ions sorption was investigated by Razzaz et al.^54^.

However, little research has focused on the TiO_2_–chitosan nanocomposite ability for COD removal from industrial wastewater. Therefore, this work applies the TiO_2_–chitosan nanocomposite as an efficient medium for COD removal from industrial wastewater (Bouali Sina Petrochemical Company, Iran). The characteristics of TiO_2_, chitosan, and fabricated TiO_2_–chitosan nanocomposites have been determined using FESEM, XRD, and FTIR tests. The BBD (Box–Benkhen design of experiment) investigates the effect of adsorption parameters (i.e., temperature, pH, contact time, TiO_2_–chitosan mass ratio adsorbent content, and solution volume) on the COD removal from the wastewater. Moreover, the optimum operating condition that maximizes the COD removal from the industrial wastewater using the TiO_2_–chitosan nanocomposite has been determined. The best kinetic and isotherm models for describing the transient and equilibrium COD removal measurements have been introduced, and their associated parameters are accurately adjusted.

## Laboratory phase

### Materials

Chitosan (molecular weight = 100 kDa, 99% degree of deacetylation), and TiO_2_ nanoparticles have been bought from Sigma-Aldrich, USA. Acetic acid and sodium chloride have been purchased from Fluka, Germany. All experimentations have been done using distilled water.

### Fabricating theTiO_2_–chitosan nanocomposite

The TiO_2_–chitosan nanocomposite was synthesized based on the procedure described by Zainal et al.^55^. Briefly, 2.5 g chitosan nanoparticles were dissolved in 40 mL NaCl (molarity = 0.2) and 30 mL acetic acid (molarity = 0.1) under 12 h of stirring. Then, 2.5 g, 1.25 g, or 5 g TiO_2_ powder (depending on the composite content, i.e., 1:1, 1:2, or 2:1) and 50 mL acetic acid (molarity = 0.1) were added to the previous solution and mixed for more than 24 h until a homogenous solution of TiO_2_–chitosan was reached. Finally, the solution was warm-up in an oven at 100 °C for 4 h until the solvent was completely evaporated and the TiO_2_–chitosan composite synthesized. Several composites with different mass ratios of TiO_2_ and chitosan (i.e., 1:1, 1:2, and 2:1) have been fabricated in the same way as described before.

### Characterization tests

This study characterizes the morphology of TiO_2_ nanoparticles, chitosan, and fabricated TiO_2_–chitosan nanocomposites applying the FESEM (field-emission scanning electron microscopy, MIRA3TESCAN-XMU) after gold coating. The functional groups of TiO_2_, chitosan, and TiO_2_–chitosan nanocomposites have been monitored utilizing the FTIR test (Fourier transform infrared spectroscopy, Perkin–Elmer Spectrum GX FTIR spectrometer). The Philips instrument (X’pert diffractometer) has been employed for recording the XRD (X-ray powder diffraction) profiles of chitosan, TiO_2_, and TiO_2_–chitosan composites at 25 °C (using CuKα radiations).

### Determination of COD of solutions

The performance of the fabricated TiO_2_–chitosan nanocomposites for reducing the wastewater COD was measured using the standard procedure of HACH. Indeed, the closed reflux method^56^ in a HACH COD reactor (DRB200, Hach Co., Loveland) containing K_2_Cr_2_O_7_ (potassium dichromate) reagent has been applied to measure the wastewater COD ranging from zero to 1500 mg L^−1^. Then, 2 mL aliquots were added to the COD vials at 150 °C for 2 h. The COD vials were cooled to room temperature and titrated with ferrous ammonium sulfate (molarity = 0.05). The solution pH adjusts using H_2_SO_4_ (molarity = 0.1) or NaOH (molarity = 0.1). After conducting the adsorption tests, the adsorbent separates from the extract through 10 min of centrifugation at 4000 rpm (Denley BS400 machine, UK). The TDS (total dissolved solids), initial pH, and COD of Bouali Sina Petrochemical Company wastewater are 574 mg L^−1^, 7.3, and 0.97 g L^−1^, respectively. Equation (1) expresses the mathematical formulation of the COD removal^57^.

1 $${\text{COD }}\;{\text{removal}}\;\left( \% \right) \, = \frac{{C_{i} - C_{o} }}{{C_{i} }} \times 100$$

where C_i_ and C_o_ stand for the initial and final COD concentrations, respectively.

### Design of experiments for the adsorption test

The current research applies the four-factor three-level BBD (Box–Behnken design) scenario to investigate the impact of adsorption parameters [i.e., adsorbent content (0.25–2.5 g), contact time (30–180 min), pH (3–11), and solution volume (100–500 mL)] on industrial wastewater COD removal using TiO_2_–chitosan nanocomposites. The polynomial model for correlating the COD removal to the adsorption parameters is defined by Eq. (2) ^58^.

2 $$COD \, removal\,\left( \% \right)\, = A_{0} + \sum\limits_{k = 1}^{4} {A_{k} \,X_{k} } + \sum\limits_{i = 1}^{4} {A_{kk} \,X_{k}^{2} } + \sum\limits_{k = 1}^{4} {\sum\limits_{z = 1}^{4} {A_{kz} \,X_{k} } } \,X_{z}$$

where A_0_, A_k_, A_kk_, A_kz_ are the model’s coefficients. X_k_, X_k_^2^, and X_k_ X_z_ are the three combinations of the independent variables (linear, quadratic, and interactive). Table 1 summarizes the output of applying the design of the experiment to the adsorption parameters. This table also reports the experimentally-measured COD removal values (see “Effect of operating conditions on the COD removal” section), and their counterpart predicted values by a polynomial model (see “Statistical analyses of the adsorption experiments” section).

**Table 1:** Summary of the design of experiment, experimental and predicted COD removal values.

Run	Independent variables				COD removal (%)	
	Solution pH	Contact time (min)	Adsorbent mass (g)	Effluent volume (mL)	Experimental	Model prediction
1	3	30	0.250	100	36.5	36.8
2	11	30	0.250	100	44.1	43.8
3	3	180	0.250	100	50.9	50.0
4	11	180	0.250	100	58.3	58.7
5	3	30	2.500	100	56.0	55.8
6	11	30	2.500	100	62.2	62.2
7	3	180	2.500	100	70.3	70.8
8	11	180	2.500	100	78.9	78.9
9	3	30	0.250	500	16.5	16.2
10	11	30	0.250	500	22.3	22.8
11	3	180	0.250	500	30.5	30.9
12	11	180	0.250	500	38.7	38.6
13	3	30	2.500	500	34.5	34.6
14	11	30	2.500	500	39.2	39.8
15	3	180	2.500	500	50.8	50.9
16	11	180	2.500	500	57.8	57.9
17	3	105	1.375	300	47.5	47.5
18	11	105	1.375	300	55.1	54.5
19	7	30	1.375	300	58.9	58.9
20	7	180	1.375	300	75.2	74.6
21	7	105	0.250	300	59.6	60.2
22	7	105	2.500	300	80.5	79.4
23	7	105	1.375	100	78.1	78.2
24	7	105	1.375	500	58.1	57.4
25	7	105	1.375	300	69.3	69.5
26	7	105	1.375	300	68.5	69.5
27	7	105	1.375	300	69.1	69.5

### Kinetic and isotherm models

In the optimum condition of the adsorption process, the temperature effect (20–50 °C) on the COD reduction of the considered industrial wastewater was also investigated. The performance of TiO_2_, chitosan, and synthesized nanocomposites with different mass ratios of 1:1, 1:2, and 2:1 TiO_2_ and chitosan on the COD reduction was examined. Two famous kinetic models [i.e., pseudo-1st-order (Eq. 3)^59^ and pseudo-2nd-order (Eq. 4)^60^] have been applied to describe the transient behavior of the wastewater COD removal using the TiO_2_–chitosan nanocomposite.

3 $$q_{t} = q_{e} \, \times \,(1 - {\text{e}}^{{ - k_{1} t}} )$$

4 $$q_{t} = k_{2} q_{e}^{2} t/\left( {1 + k_{2} q_{e} t} \right)$$

where q_e_ and q_t_ present the COD removal capacity of an adsorbent at the equilibrium state and time t, respectively.

Freundlich, Redlich-Peterson, and Langmuir isotherms have also been checked to model the equilibrium measurements of the COD removal.

## Results and discussion

### Adsorbent characterization tests

#### FESEM

**Figure 1:**
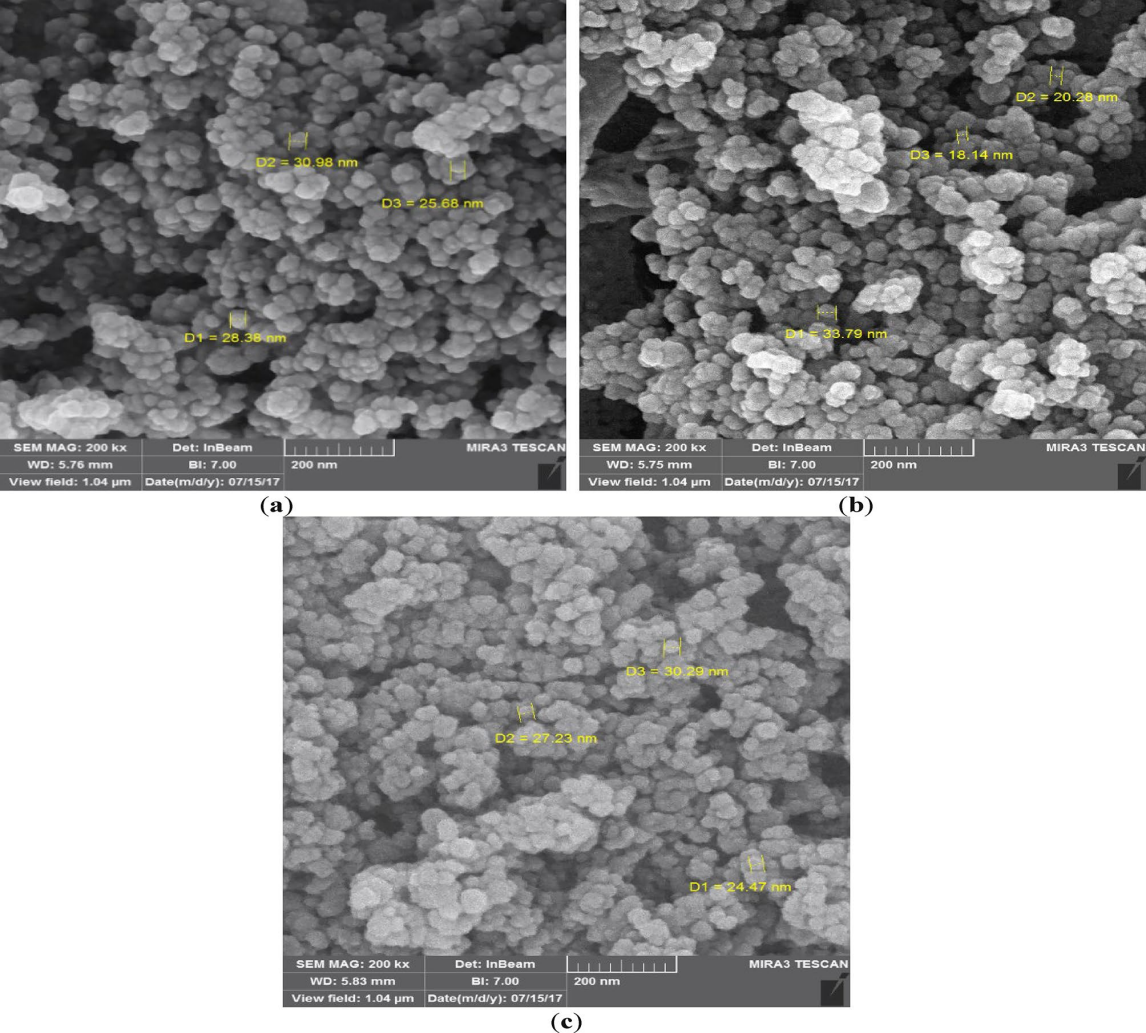
FESEM images of (**a**) TiO_2_, (**b**) chitosan, and (**c**) TiO_2_–chitosan nanocomposite.

The FESEM images of the TiO_2_ nanoparticles, chitosan, and TiO_2_–chitosan nanocomposite are presented in Fig. 1a–c, respectively.

These characterization tests show that the TiO_2_, chitosan, and TiO_2_–chitosan nanocomposite are homogenous and have an average particle size of 30, 35, and 40 nm. It can also be seen that the chitosan and TiO_2_ were appropriately dispersed in the structure of the TiO_2_–chitosan nanocomposite. The morphology of the TiO_2_–chitosan has a particle size ranging from 15 to 60 nm.

#### XRD

**Figure 2:**
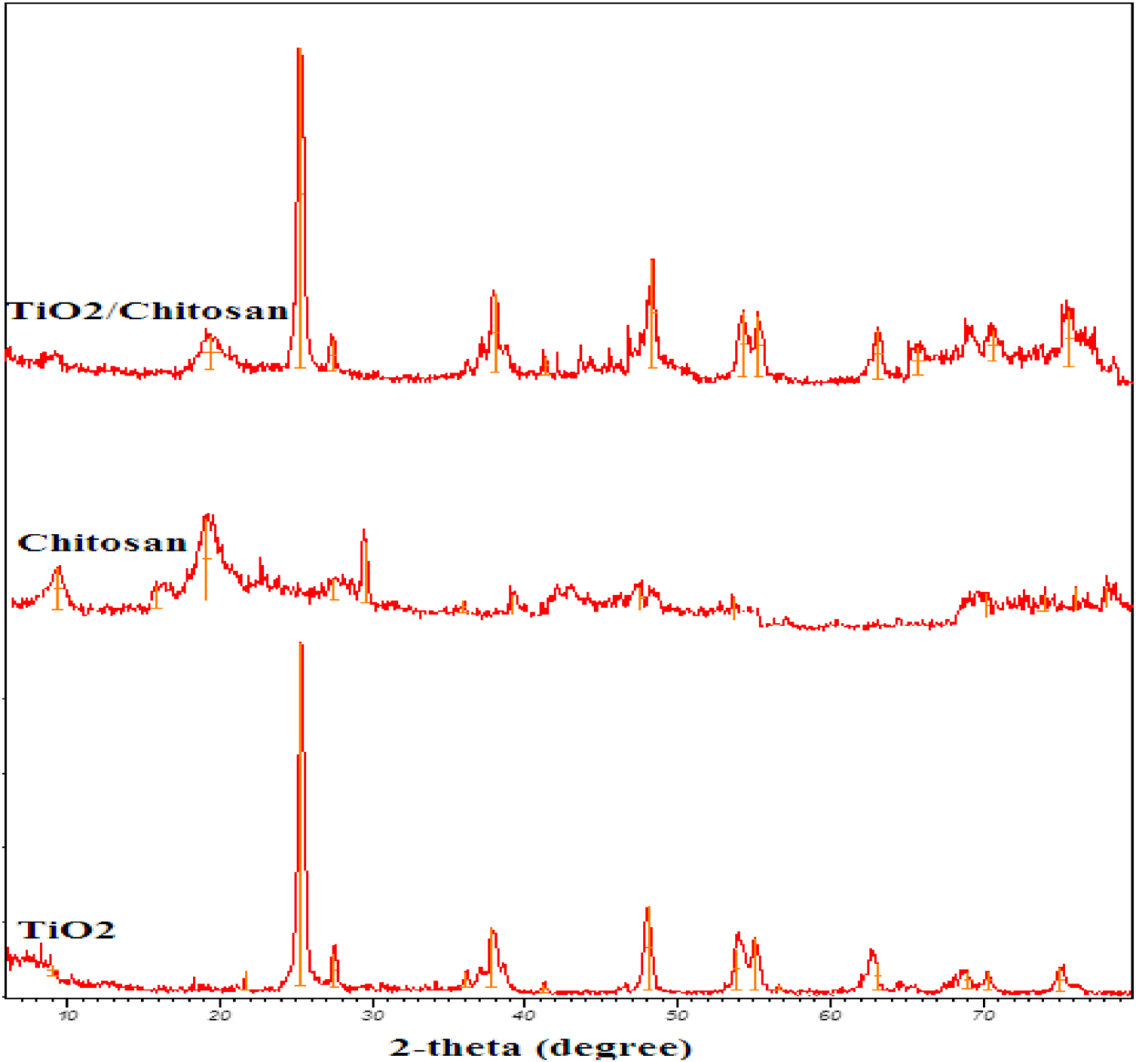
XRD patterns of TiO_2_, chitosan, and TiO_2_–chitosan nanocomposite.

The XRD patterns of chitosan, TiO_2_, and TiO_2_–chitosan nanocomposite have been displayed in Fig. 2. The peaks observed at the 2θ = 25.3° (1 0 1), 48.1° (2 0 0), 56.6° (2 1 1), 62.7° (2 0 4), and 75.1° (2 1 5) could be related to the various diffraction planes of anatase form of the TiO_2_ nanoparticles. Whereas the peaks appeared at the 2θ = 27.5°, 37.0°, 54.3°, and 70.3° correspond to the various diffraction planes of nanoparticle rutile form^61^. The peaks corresponding to chitosan’s crystalline form appear at 2θ = 10° and 19.5°. The XRD pattern of TiO_2_–chitosan nanocomposite shows that the synthesized TiO_2_–chitosan nanocomposite possesses a crystallized form with peaks at 2θ = 19.2°, 25.3°, 48.1°, 62.7°, and 75°. Comparing the XRD patterns of TiO_2_–chitosan nanocomposite and TiO_2_ indicates the presence of chitosan peaks in the TiO_2_–chitosan nanocomposite structure. Moreover, no significant change in the anatase and rutile forms of the TiO_2_ nanoparticles has occurred. This observation approved that the TiO_2_–chitosan synthesis procedure maintains the characteristic structure of TiO_2_ nanoparticles.

#### FTIR

**Figure 3:**
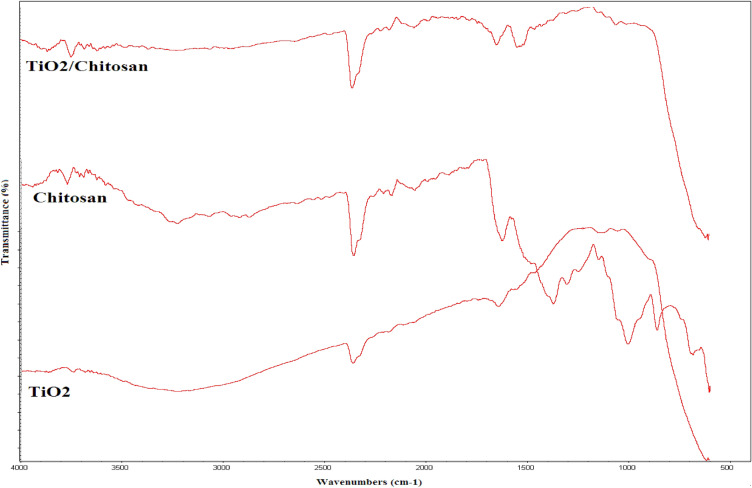
FTIR spectra of chitosan, TiO_2_ and TiO_2_–chitosan nanocomposite.

The FTIR spectra of TiO_2_ nanosized particles, chitosan, and TiO_2_–chitosan nanocomposite have been shown in Fig. 3. The absorption bands at 3720, and 1650 cm^−1^ are related to O–H and N–H groups of the polysaccharide. The stretching band at 1560 cm^−1^ could be associated with the amide content in the chitosan structure. The observed bond at 2924 cm^−1^ corresponds to the CH_2_ stretching groups. The observed bond at 2359 cm^−1^ shows the stretching carboxyl groups of chitosan. The absorption band around 1150 cm^−1^ describes the C–OH stretching vibration. The chitosan C–O stretching groups are detected at 1005 and 862 cm^−1^.

In the TiO_2_ nanoparticles’ FTIR spectrum, the absorption spectra at 3737, 3231, 2359, and 1642 cm^−1^ are associated with the hydroxyl groups. The observed band at 650 cm^−1^ revealed the existence of the TiO_2_ compound.

Moreover, the characteristic bands of the chitosan and TiO_2_ can be easily detected in the TiO_2_–chitosan nanocomposite’s FTIR spectrum. No significant differences are observable in the FTIR spectra of the chitosan, TiO_2_, and the synthesized TiO_2_–chitosan nanocomposite. It implies that the TiO_2_ addition into the chitosan structure produces no changes in the chitosan chemical structure. These observations approved that the TiO_2_ was physically loaded in the chitosan structure.

### Effect of operating conditions on the COD removal

**Figure 4:**
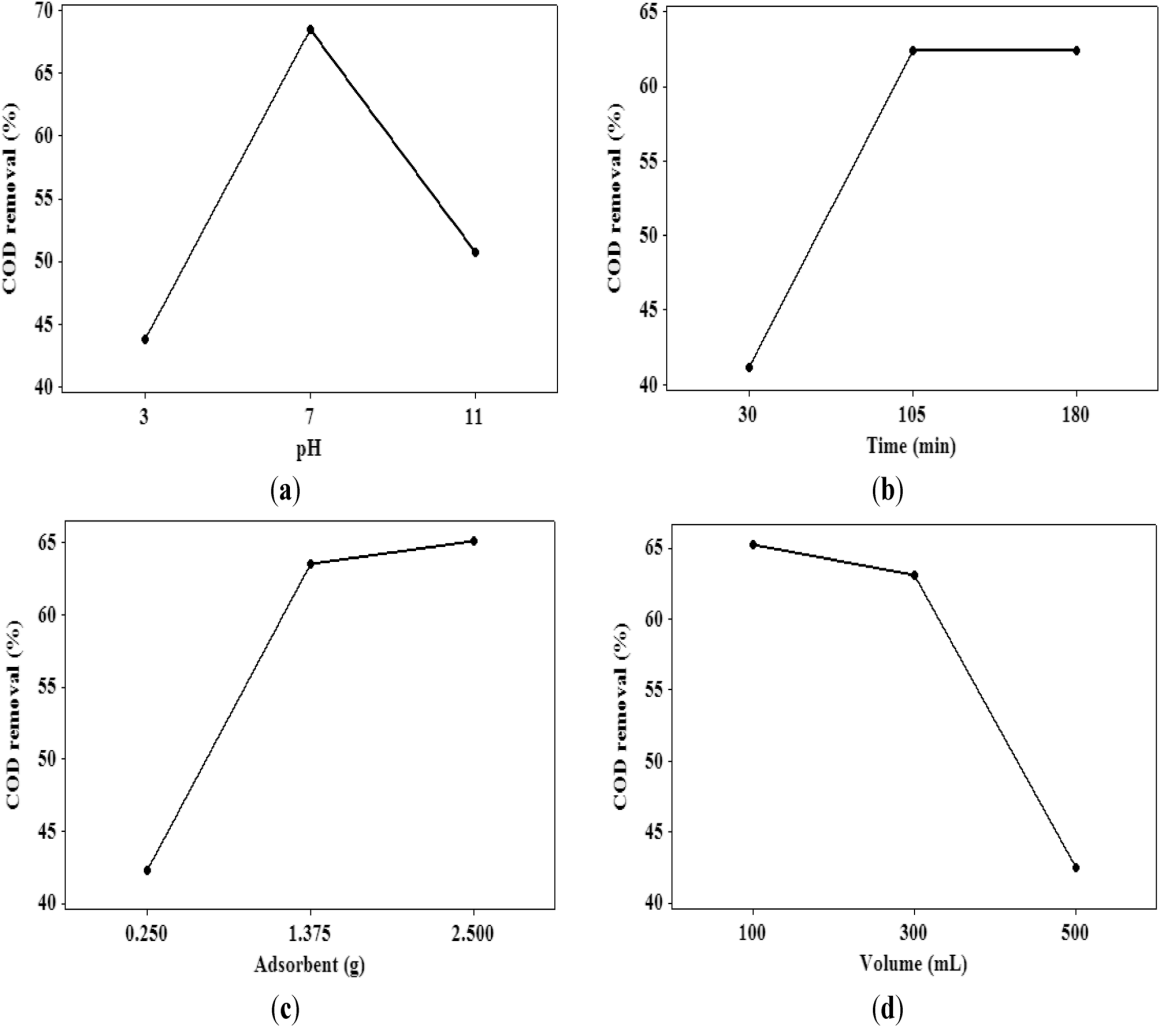
The influence of (**a**) solution pH, (**b**) wastewater-adsorbent contact time, (**c**) adsorbent content, and (**d**) solution volume on the COD removal ability of the TiO_2_–chitosan nanocomposite.

The effects of four influential factors (i.e., adsorbent content, contact time, pH, and solution volume) on the industrial wastewater COD removal have been measured at three working levels. Figure 4a shows the pH impact on the COD removal efficiency of the TiO_2_–chitosan nanocomposite. This figure states that increasing the solution pH up to 7 increases the COD removal, and after that, the COD removal efficiency of TiO_2_–chitosan nanocomposite decreases. Higher H^+^ ion concentration in the acidic solution (pH of lower than 7) neutralizes the negative charge of the TiO_2_–chitosan surface and reduces the COD removal efficiency by the ion exchange. On the other hand, high OH^-^ ion concentration in the alkaline/basic solution (pH of higher than 7) prevents the diffusion of organic materials into the TiO_2_–chitosan pores and decreases the COD removal^62^. Furthermore, the surface charge of the adsorbent depends on the solution pH. The zero charge point of a TiO_2_ in water is at pH ~ 6. At the alkaline range of pH, the positive surface charge of the adsorbent may be responsible for decreasing the COD removal efficiency of the TiO_2_–chitosan nanocomposite^40^. Similar results were also reported by other researchers^63,64^. The optimum pH value of 7 has been reported for maximizing the COD removal efficiency of some adsorbents for wastewater treatment of the coffee^63^ and sugar^64^ processing companies.

The influence of wastewater-nanocomposite contact time at three levels on the COD removal performance of the TiO_2_–chitosan adsorbent is depicted in Fig. 4b. It can be concluded that the adsorption capacity of the TiO_2_–chitosan nanocomposite increases by increasing the contact time. COD adsorption using the TiO_2_–chitosan adsorbent experiences the equilibrium state at contact time = 180 min. More than 90% of the total COD has been adsorbed at the first 105 min of the contact time. The sharp variation of the COD removal during the first 105 min of contact time is associated with the high number of active sites available at the TiO_2_–chitosan surface. After saturating surface-active sites, the organic matters require more time to diffuse through the TiO_2_–chitosan pores and adsorb on the pore walls of the nanocomposite. After 180 min of contact time, all the internal/external active sites of the TiO_2_–chitosan nanocomposite have been occupied, and the equilibrium state is reached. A similar trend has been reported for water removal from 2-dimethylaminoethylazide using calcium chloride and NaA zeolite^65^.

Figure 4c shows the influence of adsorbent content on the COD removal from industrial wastewater. This figure explains that increasing the adsorbent content increases the available active sites for pollutant adsorption and enhances the COD removal efficiency of the utilized nanocomposite. This figure also shows that the COD removal rate decreases by increasing the adsorbent content (> 1.375 g). Indeed, decreasing the available organic matter to adsorb on the active nanocomposite sites reduces the COD removal rate of a high nanocomposite dosage.

The impact of the effluent volume of wastewater/solution on the COD removal efficiency of the fabricated nanocomposite is illustrated in Fig. 4d. This figure indicates that increasing the solution volume increases the number of organic matters, rapidly saturates the available active sites of the nanocomposite, and decreases the COD removal. The low performance of the TiO_2_–chitosan nanocomposite for efficiently removing the COD of 500 mL of effluent volume is related to the rapid saturation of adsorbent sites. Indeed, the lower COD removal efficiency achieved for the high than the low wastewater effluent volume is connected to the higher COD needed to be adsorbed/removed by the same number of active sites.

**Figure 5:**
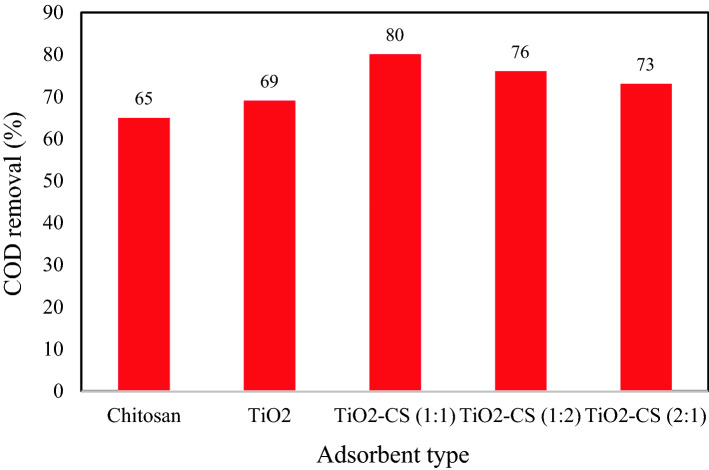
Performance of synthesized adsorbents for COD removal from wastewater (CS: Chitosan).

The COD removal ability of TiO_2_ nanoparticles, chitosan, and synthesized nanocomposites with mass ratios of 1:1, 1:2, and 2:1 of TiO_2_ and chitosan have been compared in Fig. 5. This graph shows that the maximum COD removal of 80% can be achieved by helping the TiO_2_–chitosan (1:1) adsorbent at pH 7, contact time 180 min, adsorbent content 2.5 g, and 300 mL solution volume. The COD removal ability of the utilized adsorbents has an order of TiO_2_–chitosan 1:1 (80%) > TiO_2_–chitosan 1:2 (76%) > TiO_2_–chitosan 2:1 (73%) > TiO_2_ (69%) > chitosan (65%). Therefore, the TiO_2_–chitosan with an equal mass ratio is the best adsorbent for COD removal from industrial wastewater.

**Figure 6:**
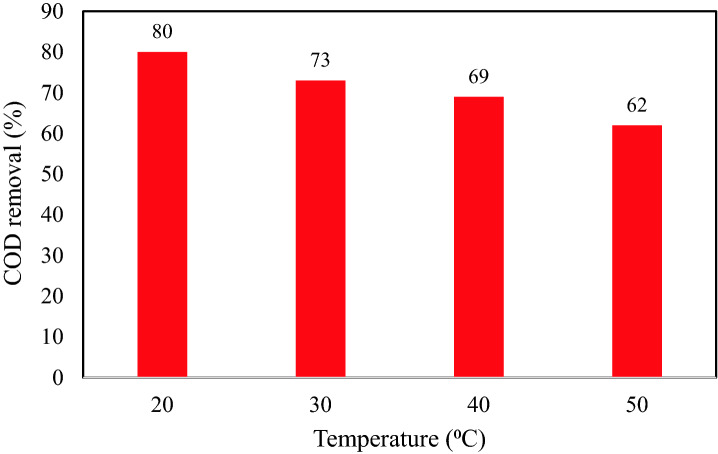
Effect of temperature on the COD reduction efficiency using TiO_2_–chitosan nanocomposite.

Dependency of the COD removal efficiency of the TiO_2_–chitosan (1:1) nanocomposite 100 mL of the effluent solution (pH 7.4, adsorbent content = 1.375 g, contact time = 105 min) has been illustrated in Fig. 6. This figure approves the negative effect of temperature on the COD removal efficiency of the TiO_2_–chitosan nanocomposite. It means that the TiO_2_–chitosan adsorbent has the highest tendency to remove the wastewater COD at low temperatures. This behavior may be associated with increasing the internal energy of pollutants that helps them to detach from the adsorbent surface and escape into the solution bulk. Exothermic adsorption may be considered the next responsible for this observation^66^. Thus, both physical and ion exchange are possible to involve in the COD sorption process using the TiO_2_–chitosan nanocomposite. This observation has also been reported by other scientists^67,68^.

### Statistical analyses of the adsorption experiments

Table 2 summarizes the results of ANOVA (analysis of variance) performed to inspect the significance probability (p-value) of influential variables on the COD removal efficiency of the nanocomposite. Those independent variables with p < 0.05 at the 95% confidence interval significantly impact the COD removal^69^. The significant variables are necessary to include in the full quadratic model^70^. On the other hand, the insignificant variables (p > 0.05) should be removed from the full quadratic model^71^.

**Table 2:** ANOVA outcomes for monitoring wastewater COD removal using the TiO_2_–chitosan nanocomposite.

Source	DF	Seq SS	F	p-value
Regression	14	7721.83	1078.67	0.000
Linear	4	4928.36	2409.56	0.000
pH	1	221.20	432.60	0.000
Contact time (min)	1	1107.64	2166.17	0.000
Adsorbent mass (g)	1	1658.88	3244.22	0.000
Effluent volume (mL)	1	1940.65	3795.26	0.000
Square	4	2783.37	1360.84	0.000
pH × pH	1	2737.78	1724.92	0.000
Contact time × contact time (min^2^)	1	37.78	38.60	0.000
Adsorbent mass × adsorbent mass (g^2^)	1	0.19	0.27	0.616
Effluent volume × effluent volume (mL^2^)	1	7.61	14.88	0.002
Interaction	6	10.10	3.29	0.037
Contact time × pH (min)	1	2.98	5.82	0.033
adsorbent mass × pH (g)	1	0.39	0.76	0.399
pH × effluent volume (mL)	1	1.05	2.05	0.177
Adsorbent mass × contact time (g min)	1	2.98	5.82	0.033
Contact time × effluent volume (min mL)	1	2.03	3.97	0.070
Adsorbent mass × effluent volume (g mL)	1	0.68	1.33	0.271
Residual error	12	6.14		
Lack-of-fit	10	5.79	3.34	0.252
Pure error	2	0.35		
Overall	26	7727.97		

Table 3 reports the ANOVA results for only the significant variables (p < 0.05). Equation (5) presents the polynomial model developed to predict COD removal from the significant variables.

5 $$COD \,\,removal\,\left( \% \right)\, = \, - 7.46538\, + \,16.87337\,X_{1} \, + \,0.18848X_{2} \, + \,7.99667\,X_{3} \, - \,0.027095\,X_{4} \, - \,1.15342\,\,X_{1}^{2} - 0.000481\,X_{2}^{2} \, - \,0.000041\,X_{4}^{2} \, + \,0.001437\,X_{1} \,X_{2} \, + \,0.005111\,X_{2} \,X_{3}$$

where X_1_, X_2_, X_3_, and X_4_ stand for the solution pH, contact time (min), adsorbent content (g), and effluent solution volume (mL), respectively. Comparing the lack of fit before (0.252) and after (0.224) elimination of the insignificant parameters reveals considerable improvement in the model prediction accuracy. A relatively high achieved correlation coefficient (R^2^ > 0.99) implies an excellent compatability between the experimental COD removal values and their counterpart predictions by the developed model. Equation (6) presents the mathematical form of the R^2^ ^72^.

6 $$R^{2} = \,1 - \sum\limits_{i = 1}^{N} {\left( {COD^{\exp } - COD^{cal} } \right)_{i}^{2} /\sum\limits_{i = 1}^{N} {\left( {COD^{\exp } - \overline{{COD^{\exp } }} } \right)_{i}^{2} } } \,$$

**Table 3:** Summary of the ANOVA results after eliminating the insignificant independent variables.

Source	DF	Seq SS	F	p-value
Regression	9	7717.54	1398.45	0.000
Linear	4	4928.36	2009.34	0.000
pH	1	221.20	360.74	0.000
Contact time (min)	1	1107.64	1806.37	0.000
Adsorbent mass (g)	1	1658.88	2705.36	0.000
Effluent volume (mL)	1	1940.65	3164.88	0.000
Square	3	2783.23	1513.00	0.000
pH × pH	1	2737.78	1555.20	0.000
Contact time × contact time (min^2^)	1	37.78	33.41	0.000
Effluent volume × effluent volume (mL^2^)	1	7.67	12.50	0.003
Interaction	2	5.95	4.85	0.022
pH × time (min)	1	2.98	4.85	0.042
Contact time × adsorbent mass (min g)	1	2.98	4.85	0.042
Residual error	17	10.42		
Lack-of-fit	15	10.08	3.88	0.224
Pure error	2	0.35		
Overall	26	7727.97		

### Validation of the model performance

**Figure 7:**
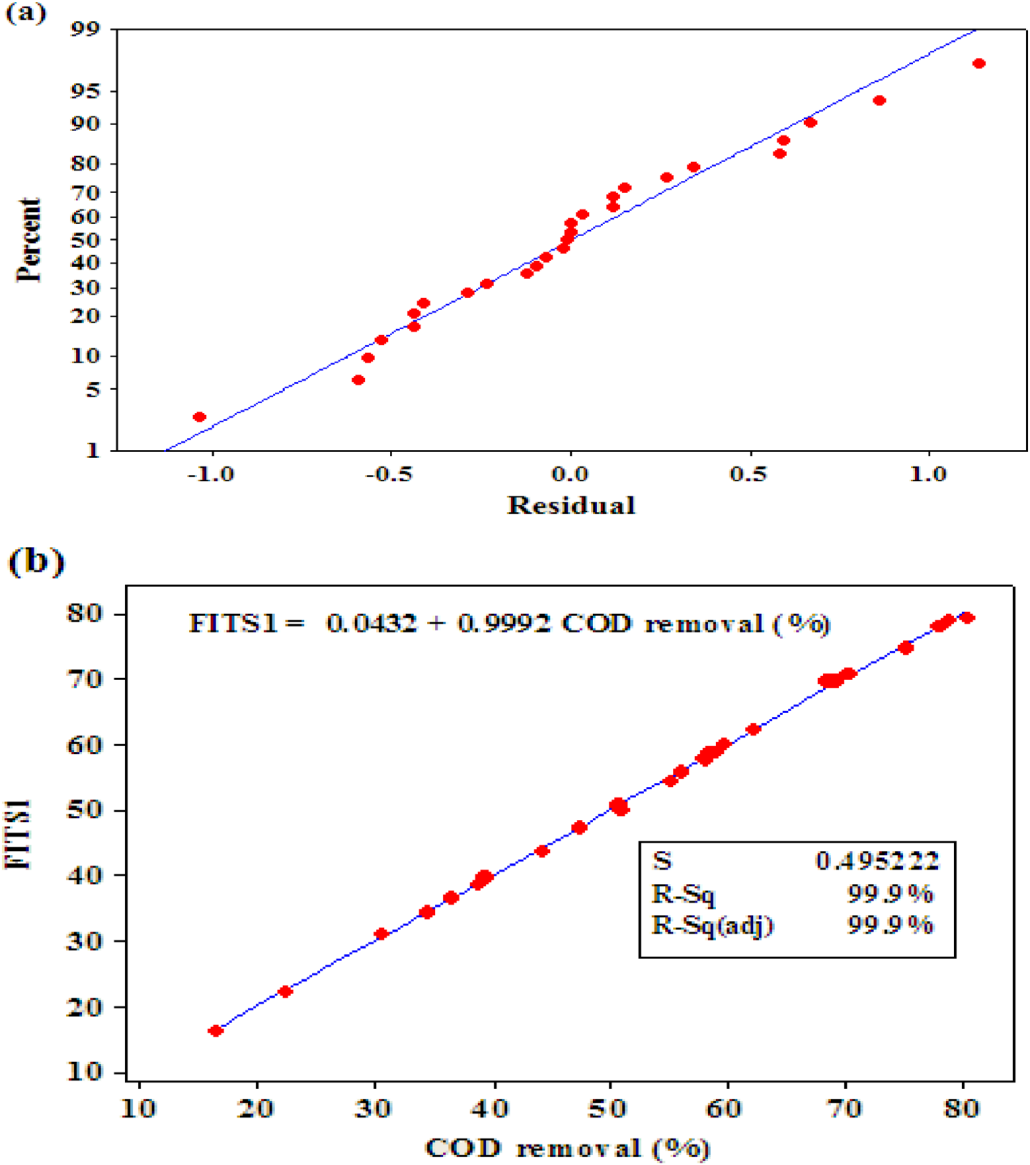
(**a**) the normal probability graph of residuals and (**b**) cross-plot of the experimental and predicted COD removal values.

A normal probability graph of residuals is depicted in Fig. 7a. This figure states that all data samples have approximately located around the straight diagonal line. Based on the Yetilmezsoy et al. observation, errors have a normal distribution and are independent of each other^73^. The cross-plot of the predicted COD removal (FITS1) versus their associated experimental measurements has been exhibited in Fig. 7b. It can be easily concluded that slight deviations exist between the experimental COD removal values and the model predictions. The high correlation coefficient value (R^2^ = 0.999) approves that the constructed model accurately approximates the experimentally-measured COD data.

### Optimizing the adsorption parameters

It is possible to locate the optimized values of the involved independent variables by solving Eq. (5). The optimum values of the solution pH, contact time, adsorbent content, and effluent solution volume are 7.4, 180 min, 2.5 g, and 100 mL, respectively. In this optimum condition, the maximum COD removal efficiency of the TiO_2_–chitosan nanocomposite is 93.67%. The experimental value of the COD removal under the optimum condition (i.e., 94.5%) is also in excellent agreement with the predicted optimized value.

### Monitoring the combined effect of independent variables

**Figure 8:**
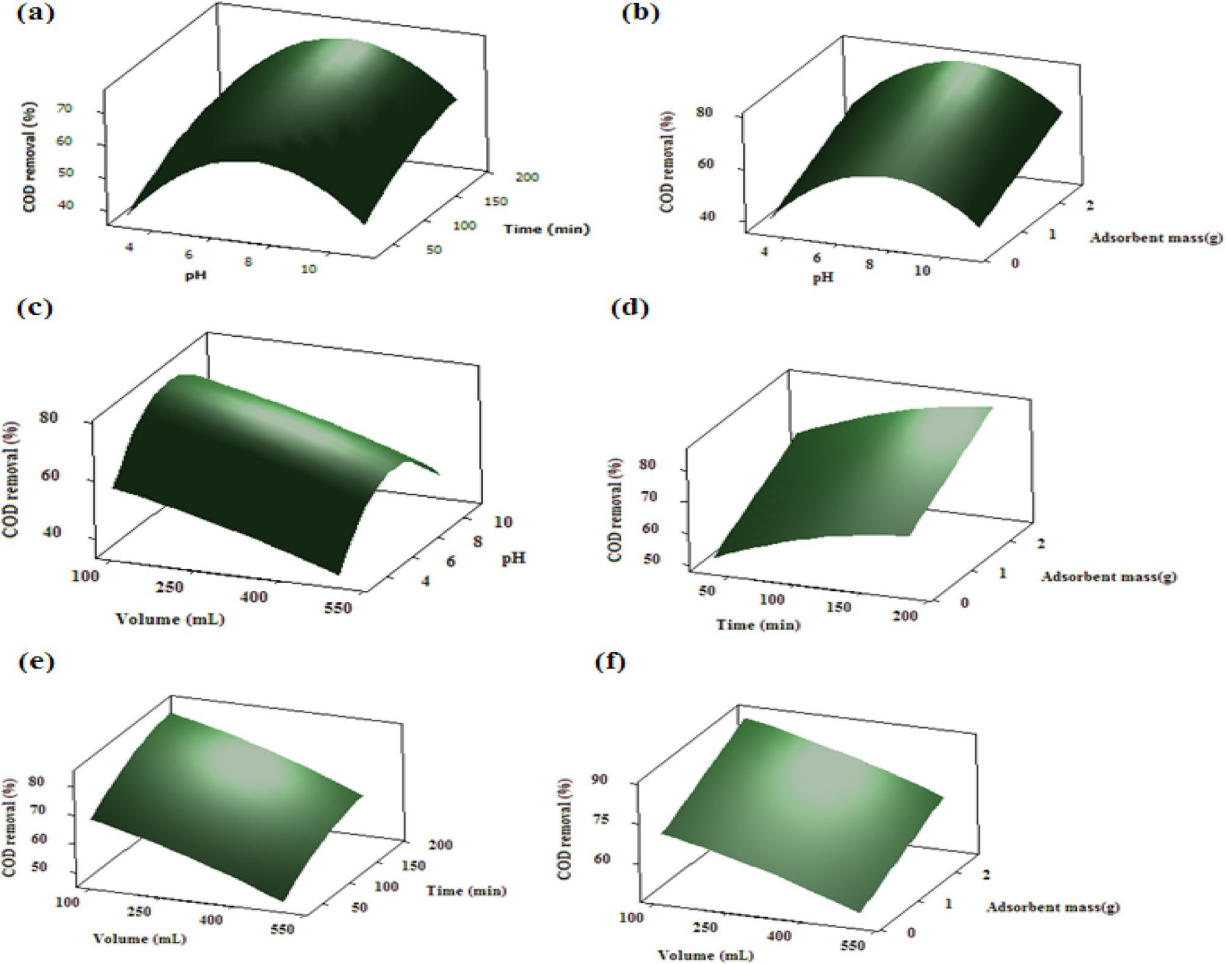
Variation of the COD removal by the (**a**) solution pH–time, (**b**) solution pH–nanocomposite mass, (**c**) pH–wastewater volume, (**d**) time–nanocomposite mass, (**e**) time–wastewater volume, and (**f**) nanocomposite mass–wastewater volume.

Figure 8 exhibits several three-dimensional (3D) graphs for monitoring the dependency of the COD removal ability of the TiO_2_–chitosan nanocomposites on different combinations of influential variables. Each of these figures explains the combined effect of a pair of independent variables on the COD removal (the central level of the other two variables has been used to plot these figures). The surface plot of the estimated COD removal as a function of pH and contact time has been shown in Fig. 8a. The COD removal efficiency of the TiO_2_–chitosan nanocomposite increases by increasing the solution pH up to 7 and then decreases at higher pH values. This behavior was previously related to the variation of the adsorbent surface charge by the solution pH. Increasing the COD removal over time is related to the greater period available for organic matter to absorb on the nanocomposite surface and diffuse in its pores. The simultaneous effects of pH and adsorbent content (Fig. 8b) and pH and effluent solution volume (Fig. 8c) on the wastewater COD removal reveal that the optimum pH value is about 7. The couple effects of pH and contact time (Fig. 8a), adsorbent dosage and contact time (Fig. 8d), and contact time and volume solution (Fig. 8e) indicate that two different mechanisms govern the COD adsorption efficiency of the utilized nanocomposite over the time. In the first stage (up to 105 min), the fast COD adsorption may be related to the adsorption of organic matters on the external surface of the TiO_2_–chitosan nanocomposite. In the second stage, the organic matter diffuses through the TiO_2_–chitosan composite pores and throats and absorbs on the internal active sites.

Furthermore, enhancing the COD removal by the TiO_2_–chitosan nanocomposite dosage can be related to increasing the available surface area and active sites for adsorbing pollutants (Fig. 8b,d,f). The simultaneous effect of volume and pH, adsorbent dosage, and contact time have been presented in Fig. 8c,e,f. These graphs state that increasing the effluent wastewater volume negatively affects the COD removal efficiency of the nanocomposite. An increase in the effluent wastewater volume increases the pollutant concentration, rapidly saturates the active sites, and reduces the COD removal ability of the nanocomposite.

### Kinetic studies

Referring to Eqs. (3) and (4), the adjustable constants of the pseudo-1st-order and pseudo-2nd-order kinetic equations are shown by k_1_ and k_2_, respectively. Table 4 introduces the adjusted constants of the considered kinetic models, experimental and calculated values of the COD adsorption capacity at the equilibrium state, and the observed correlation coefficients. Since the pseudo-2nd-order has a higher correlation coefficient than that of the pseudo-1st-order kinetic approach, the earlier better describes the transient behavior of the COD removal by the TiO_2_–chitosan nanocomposite. Furthermore, the COD adsorption capacity obtained by the pseudo-2nd-order has a higher compatibility with the experimental measurements (R^2^ = 0.993) than those provided by the pseudo-1st-order kinetic model (R^2^ = 0.970). Thus, the pseudo-2nd-order kinetic model is chosen for modeling the transient behavior of the wastewater COD removal using the TiO_2_–chitosan adsorbent. This is close to the results discussed by Prakash et al.^35^.

**Table 4:** Kinetic parameters of COD sorption using TiO_2_–chitosan adsorbent.

Adsorbent	q_exp_	Kinetic model					
		Pseudo-1st-order			Pseudo-2nd-order		
TiO_2_–chitosan	9.75	q_e_	k_1_	R^2^	q_e_	k_2_	R^2^
		9.32	0.0283	0.970	9.85	0.00106	0.993

### Isotherm models

Three well-established isotherm models, namely Freundlich^74^, Redlich–Peterson^75^, and Langmuir^76,77^, have been employed to monitor the equilibrium behavior of the COD removal performance of the TiO_2_–chitosan nanocomposite. The mathematical form of Freundlich [Eq. (7)], Redlich–Peterson [Eq. (8)], and Langmuir [Eq. (9)] isotherms has been shown below.

7 $$q_{e} = k_{F} C_{e}^{1/n}$$

8 $$q_{e} = PC_{e} /\left( {1 + \alpha C_{e}^{\beta } } \right)$$

9 $$q_{e} = q_{m} bC_{e} /\left( {1 + bC_{e} } \right)$$

where k_F_ and n are Freundlich’s model constants. q_m_ and b show the coefficients of the Langmuir model. P, α, and β stand for the Redlich–Peterson model parameters. Adjusted parameters of the selected isotherms for describing the equilibrium COD removal using the TiO_2_–chitosan composite have been reported in Table 5. This table also introduces the numerical values of the observed correlation coefficients. It can be seen that the Redlich–Peterson isotherm model has the highest R^2^ value (i.e., 0.991), and the Freundlich isotherm possesses the smallest R^2^ (i.e., 0.970). Since the adjusted value of the β (for the Redlich–Peterson isotherm) is close to 1, it can be concluded that the monolayer COD adsorption by the TiO_2_–chitosan nanocomposite is the predominant scenario.

**Table 5:** Isotherm parameters for COD removal applying the TiO_2_–chitosan composite.

Adsorbent	Freundlich			Langmuir			Redlich–Peterson			
TiO_2_–chitosan	k_F_	n	R^2^	q_m_	k_L_	R^2^	P	α	β	R^2^
	38.5	5.236	0.970	89.5	1.005	0.982	5.92	0.0621	0.939	0.991

### Cost analysis

The cost of chitosan and TiO_2_ nanopowder was obtained as presented in Table 6. According to Table 6, maximum production cost of nano composite TiO_2_–chitosan (1:1) is about 2.96 $ per kg of adsorbent.

**Table 6:** Cost of chitosan and TiO_2_ nano-powder.

Material	Purity	Company	Country	Price ($/kg)	Reference
Chitosan	≥ 85%	–	China	1.00–2.00	www.alibaba.com
	> 99%	Wuhan Yingnuo Technology	China	0.4–2.4	https://wuhanyingnuo.en.made-in-china.com/
TiO_2_	98%	XUELIAN	China	2.00–3.30	www.alibaba.com
	≥ 96.5%	LEITAI	China	2.15–3.53	www.alibaba.com

## Conclusion

This research studied industrial wastewater treatment using TiO_2_ nanoparticles, chitosan, and TiO_2_–chitosan nanocomposite from experimental and numerical points of view. The considered adsorbents have been characterized by the XRD, FTIR, and FESEM tests. The XRD pattern proved that the synthesized TiO_2_–chitosan nanocomposite preserves the characteristic structure of TiO_2_ nanoparticles. Analyzing the FTIR spectra approved that the TiO_2_ nanoparticles have been physically loaded in the chitosan structure. The FESEM tests confirmed that the TiO_2_–chitosan nanocomposite has a particle size ranging from 15 to 60 nm. The impact of solution pH, temperature, adsorbent mass and composition, contact time, and effluent solution volume on the COD removal has been monitored using the experimental and modeling analyses. The optimum condition for the considered process (pH 7.4, contact time = 180 min, nanocomposite mass = 2.5 g, and wastewater effluent volume = 100 mL) has been determined using the Box–Behnken design of the experiment. Furthermore, results approved that the TiO_2_–chitosan (1:1) at the lowest allowable temperature is better to employ for industrial wastewater treatment. The maximum experimental and calculated COD removal efficiency of the TiO_2_–chitosan nanocomposite is 93.67% and 94.5%, respectively. The Redlich–Peterson isotherm and Pseudo-2nd-order kinetic models showed the best performances for describing the equilibrium and kinetic measurements of the wastewater COD removal by the fabricated nanocomposite.

## Data Availability

All data generated or analyzed during this study are available on reasonable request from the corresponding author.
